# Increased expression of ARF GTPases in prostate cancer tissue

**DOI:** 10.1186/s40064-015-1136-y

**Published:** 2015-07-12

**Authors:** Claire Morgan, Paul D Lewis, Lynda Hopkins, Stephanie Burnell, Howard Kynaston, Shareen H Doak

**Affiliations:** Institute of Life Science, Swansea University, Swansea, UK; Respiratory Diagnostics group, Institute of Life Science 2, Swansea University, Swansea, UK; Pathology Department, Singleton Hospital, Swansea, UK; Department of Surgery, Cardiff School of Medicine, Heath Park, Cardiff, UK

**Keywords:** Prostate cancer, ARF GTPases, Immunohistochemistry, Aggressive disease

## Abstract

**Purpose:**

ARFs are a family of Ras-related GTP binding proteins, ARF6, in particular, is implicated in cancer invasion and metastasis. However, the role of ARF proteins in prostate cancer have yet to be investigated.

**Methods:**

Immunohistochemical staining for ARF6 was performed on a prostate cancer tissue microarray with patient matched normal specimens.

**Results:**

Antibody staining was significantly over-expressed in prostate cancer patient samples compared to normal patient tissue and a trend towards increased staining intensity in cancer samples with Gleason scores of 8 and above (metastatic disease).

**Conclusion:**

Due to high homology between members of the ARF family we could not determine if ARF 6 was the only ARF over-expressed in the prostate cancer samples. However, we are the first to show that ARF-GTPases are over expressed in prostate cancer which provides further insight into the molecular biology of prostate cancer.

## Background

Prostate cancer (PCa) is the most common cancer in men (excluding non melanoma skin cancer) in the UK with over 40,000 cases diagnosed each year (CancerResearchUK 2015). However, for some men with early stage PCa that is confined to the prostate the disease may never be life threatening and can follow and indolent course which does not require urgent or invasive treatment. Unfortunately prostate specific antigen levels (PSA), Gleason score and clinical and pathological grading used to diagnose and grade PCa lack the specificity or sensitivity to distinguish between patients with indolent PCa and those requiring radical treatment. Changes in PSA can precede clinical disease progression by months or years. Thus the question of when to start therapy in PCa patients can be problematic. This poses a significant problem for clinicians when deciding the best treatment for their patients. There is, therefore, the clear need for a better understanding of the mechanisms involved in the development, progression and metastasis of prostate cancer. Key molecular targets need to be identified that discriminate normal tissue from benign tumours and benign tumours from aggressive disease and which could also function as targets of inhibition for future cancer therapeutics.

Adenosine diphosphate-ribosylation factors (ARFs) are a family of Ras-related GTP binding proteins. (Li et al. 2009). Out of the ARF GTPases, ARF 6 in particular, has received much attention; ARF6 functions are concerned with actin cytoskeletal remodelling, cell polarity and cell migration and thus may have an important role in driving carcinogenesis (Donaldson 2003). Indeed, analysis of several breast cancer cell lines of differing metastatic capacity showed a direct correlation between ARF6 protein expression and their invasive potential (Hashimoto et al. 2004). Elevated levels of activated ARF6 (ARF6-GTP) have been found to increase the invasive capacity of melanoma cells both in vitro (Tague et al. 2004) and in vivo (Muralidharan-Chari et al. 2009), while silencing ARF6 by small-interfering RNA inhibits the ability of breast cancer cells to invade through an artificial basement membrane, thereby providing evidence that ARF6 may be particularly important in driving tumour metastasis (Hashimoto et al. 2004). ARF6, therefore, may be a potential cancer biomarker, however its role in prostate carcinogenesis has yet to be investigated.

## Methods

### Immunohistochemistry

To determine whether an increased expression of ARF6 protein (suggestive of increased activity) maybe be found in prostate cancer patients we performed immunohistochemistry (IHC) on a commercially available prostate cancer tissue microarray (TMA) (AccuMax array, ISU ABXIS, Seoul, Korea). The prostate TMA contained 32 prostate cancer specimens provided in duplicate along with corresponding normal tissue. Two specimens were found to be missing in the cancer samples along with two in the normal samples bringing the final sample size to 30 cancer samples and 30 normal samples. Patient data provided with the TMA showed that the age ranged from 44 to 80 years (mean 63 years) and Gleason score ranged from 6 to 9. IHC was performed using the Benchmark XT automated staining system (Ventana, AZ) with iView^TM^ DAB detection kit (Ventana, AZ) according to the manufacturer’s instructions. After antigen retrieval, anti-ARF6 antibody [(SC-7971) Santa Cruz Biotechnology, Germany] was used at 1:50 dilution at 36°C for 36 min. Intensity of ARF6 immunostaining was scored with a four-tiered system (0–3) with 0 representing no staining, 1 weak staining, 2 moderate staining and 3 strong staining. A score for staining distribution was also applied with 0 representing no staining, 1 < 10% cellular distribution, 2 between 10 and 60% cellular involvement and 3 > 60% cellular involvement. The sum score of intensity and distribution was then determined (Jan et al. 2009).

### Statistical analysis

As IHC staining scores did not follow a normal distribution, pair-wise comparison of staining patterns between normal and cancer samples was carried out using the non-parametric Mann–Whitney U-test to determine statistical significance. A one-tailed Fisher’s Exact Test was used to determine association between staining categories in tumour and normal tissue with mid-P variant. P-values <0.05 were considered significant.

## Results

**Figure 1:**
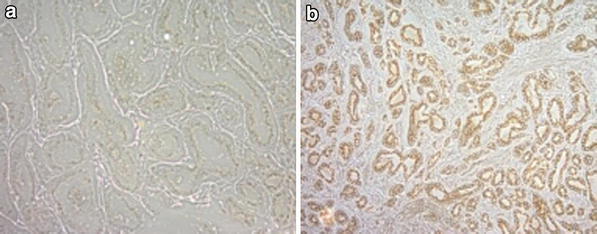
Immunohistochemical detection of ARF protein expression in **a** normal prostate tissue and **b** prostate cancer tissue scored as Gleason grade 9.

**Figure 2:**
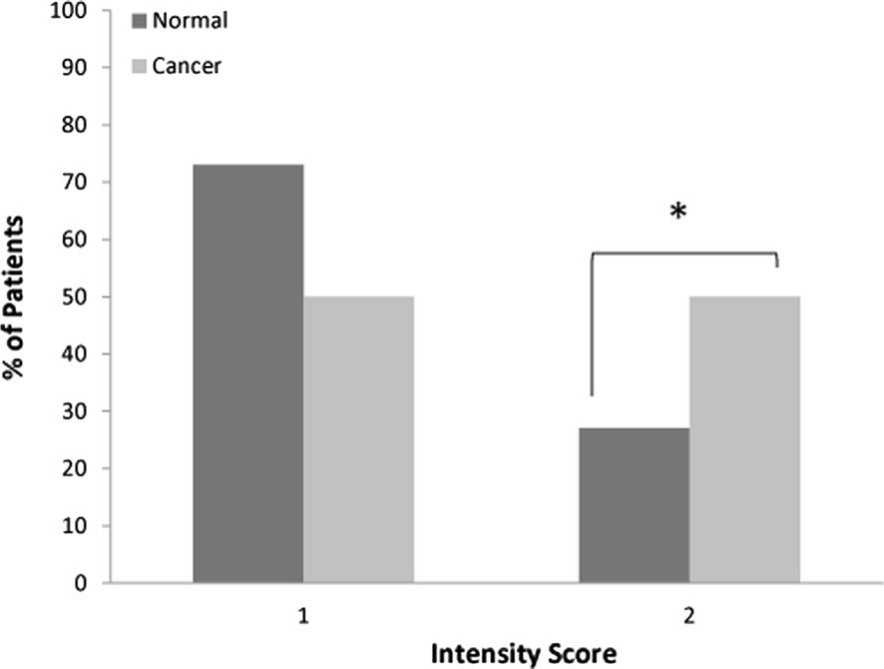
Comparison of the percentage of cancer and control patients showing, weak staining (intensity score 1) and moderate staining (intensity score 2). *Asterisk* denotes significant difference (p < 0.05) in moderate staining intensity between normal and cancer patients.

Immunohistochemical analysis using a commercial antibody to ARF6 showed a significant increase in the observed sum of staining intensity (protein expression) and distribution between cancer and matched normal samples (*P* = 0.047, Figure 1). In normal tissue 73% (N = 22) of the samples showed a higher proportion of weak staining intensity relative to moderate staining (27%, N = 8) but there were equal numbers of cases showing both weak (N = 15) and moderate staining intensity (N = 15) in tumour tissue (Figure 2). Thus, there was an observed higher level of moderate staining intensity in tumours relative to normal tissue. Based on the hypothesis of increased staining/protein expression in tumour samples, the difference was statistically significant (P = 0.044). However, there was no significant difference in staining distribution in tumour (score = 2, N = 7; score = 3, N = 23) relative to normal tissue (score = 2, N = 8; score = 3, N = 22) (P = 0.360). Thus, 77 and 73% of tumour and normal samples respectively showed ARF6 staining in >60% of the tissue sample.

Although the numbers of tumours are relatively small in this study, we made a preliminary assessment of the patterns of Gleason scores (7, 8 and 9) relative to ARF6 intensity. Gleason grade 6 was excluded from analysis due to small sample size (N = 5). There were a higher proportion of tumours with weak staining (67%, N = 10) compared to moderate staining (33%, N = 5) when Gleason score was 7. For cases with Gleason scores ≥8 the pattern is reversed with a higher proportion of moderate staining (70%, N = 7) relative to weak staining (30%, N = 3) suggesting a trend towards increased staining intensity with increasing Gleason score.

## Discussion

ARF6, a member of the ARF family of RAS related GTPases, functions in a range of biological activities such as adherens junction disassembly (Palacios et al. 2001), cell migration (Kondo et al. 2000) and cell proliferation (Li et al. 2009). ARF6 has been shown to be over-expressed in cancer cells with higher protein levels correlating with a highly invasive nature. ARF6 protein over expression was found to be between 10 and 20-fold higher in highly invasive breast cancer cell lines when compared to non-invasive and normal epithelial cells (Hashimoto et al. 2004). In an in vivo study investigating ARF6 expression, melanoma growth and metastasis, ARF6 was shown to reduce tumour growth but significantly increased the invasive capacity of the tumour cells (Muralidharan-Chari et al. 2009). However no studies have been conducted on PCa and yet there is a clear need for a better understanding of the mechanisms involved in its development, progression and metastasis. Key molecular targets need to be identified that aid diagnosis, discriminate benign tumours from aggressive disease and which could also function as targets of inhibition for future cancer therapeutics, which makes AFF6 an ideal candidate for investigation.

Our aim was to determine if ARF6 protein is over expressed in PCa samples when compared to normal prostate tissue and to correlate any over expression with Gleason score in the hope of elucidating a new PCa biomarker. It should be noted that members of the human ARF GTPase family are highly homologous with greater than 70% sequence identity between the most divergent isoforms (ARF1 and ARF6) (Haynes et al. 2007) thus we cannot rule out the possibility that the antibody to ARF6 used in this study is also detecting expression of other ARF proteins. However, our preliminary study showed ARF antibody staining to be significantly increased in PCa samples compared to normal prostate tissue with a significantly higher proportion of tumours showing moderate staining intensity compared to normal prostate tissue. In addition, a higher proportion of tumours showed weak staining intensity and thus weak protein expression compared to moderate staining when Gleason score was 7, whereas Gleason scores of 8 and above showed a higher proportion of moderate staining/protein expression relative to weak staining suggesting a trend towards increased staining intensity with increasing Gleason score.

## Conclusion

This study is the first to report the detection and over-expression of ARF proteins in prostate tissue with a significant increase in protein expression in PCa tissues compared to normal prostate tissue. While further work needs to be undertaken to determine which of the ARF proteins are over expressed, our data provides further insight into the molecular biology of prostate cancer.
